# Effect of type and amount of modified corn starches on qualitative properties of low‐protein biscuits for phenylketonuria

**DOI:** 10.1002/fsn3.1304

**Published:** 2019-12-04

**Authors:** Alireza Azaripour, Hajar Abbasi

**Affiliations:** ^1^ Department of Food Science and Technology Faculty of Agriculture Isfahan (Khorasgan) Branch Islamic Azad University Isfahan Iran

**Keywords:** biscuits, modified corn starch, phenylketonuria, physicochemical properties

## Abstract

The effect of corn starches (natural, pregelatinized, and cross‐linked) in four levels on quality of low‐protein biscuit was evaluated. First, the microscopic structure, thermal properties, and low‐branched chains content of them were evaluated. Water absorption capacity (WAC) and gelatinization properties of combined starches and physicochemical properties of products were assessed. Cross‐linked starch had the highest glass transition temperature (*T_g_*). The maximum and minimum WAC was observed in samples containing 100% pregelatinized and 100% cross‐linked starches, respectively. An increase in the amount of pregelatinized starch was accompanied by a decrease in the maximum viscosity and gelatinization temperature. Pregelatinized starch improved sensory and textural characteristics of samples, while cross‐linked starch affected baking properties of the product. The best sample containing 30% of pregelatinized and 70% cross‐linked starch with 1.27 g protein and 12 mg phenylalanine in 100 g biscuit is introduced as the low protein and safe product for Phenylketonuria.

## Graphical abstract

The effect of corn starches (natural, pre‐gelatinized and cross‐linked) in four levels on quality of low‐protein biscuit was evaluated. Cross‐linked starch had the highest glass transition temperature (*T_g_*). The maximum and minimum WAC was observed in samples containing 100% pre‐gelatinized and 100% cross‐linked starches, respectively. An increase in the amount of pre‐gelatinized starch was accompanied by a decrease in the maximum viscosity and gelatinization temperature. Pre‐gelatinized starch improves sensory and textural characteristics of samples, while cross‐linked starch affected baking properties of the product. The best sample containing 30% of pre‐gelatinized and 70% cross‐linked starch with 1.27 g protein and 12 mg phenylalanine in 100 g biscuit is introduced as the low protein and safe product for Phenylketonuria.

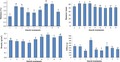

## 1 INTRODUCTION

Phenylketonuria (PKU) is a genetic disorder of phenylalanine metabolism. It is due to mutations in both copies of the phenylalanine hydroxylasegene and chromosome 1, which is responsible of making this liver enzyme. Mutations in the structure of this enzyme disturb converting phenylalanine to tyrosine and as increase the accumulation of phenylalanine in blood, neurons, and brain. In people with the normal function of the enzyme, phenylalanine converts to tyrosine and other by‐products, and therefore, the amount of blood phenylalanine remains at a normal level (Demirkol, Giżewska, Giovannini, & Walter, 2011).

Phenylalanine is one of the essential amino acids in human diet that can be found in different amounts in protein‐based foods. For Phenylketonuria patient, a controlled diet with low level of phenylalanine is required to prevent the accumulation of this amino acid in blood. In order to prevent brain and neurological disorders, diet begins immediately at birth and continues until the end of life. In the management of this metabolic disorder, a diet with a low amount of phenylalanine can improve the quality of treatments and the patients' care (Harding, 2010).

Proteins have considerable effect on the qualitative properties of cereal‐based products like their organoleptic and textural properties. Nonproteinaceous compounds with similar functional properties to proteins are suitable to improve the quality of low‐protein or protein‐free products. Potato, rice, oat, wheat, and corn starches were broadly used as the main constituents of low‐protein/protein‐free bakery products (Zannini, Kingston, Arendt, & Waters, 2013). In this regard, Yaseen and Shouk (2011) used corn, potato, cassava, and rice starches for production of protein‐free product. Phenylalanine content of the selected baked product was about 45 mg that is in the tolerable range of patients. In another study, corn starch was used to produce low‐protein biscuits for phenylketonuria. Therefore, starch is a relatively suitable substitute for a part or whole of wheat flour in various low‐protein cereal‐based products (Yaseen, 2011). Although low‐protein biscuits have been developed, a little work has been carried out on evaluating the physicochemical properties of the biscuit formulated by modified starches. Application of modified starches in the food industry can reduce their adverse effects on the quality especially appearance and texture of cereal‐based products (Koo, Lee, & Lee, 2010). Modified starches are produced by various enzymatic, physical, and chemical reactions. Pregelatinized and cross‐linked starches are two kinds of the useful modified starches (Ogura, 2004). Cross‐linked starch is widely used as thickeners, especially in products with high viscosity and stability. Cross‐linking reduces starch granule fractures in processing, past viscosity reduction, and adhesiveness of dough in baking process (Neelam, Vijay, & Lalit, 2012). Pregelatinized starch is widely used in the food industry. Pregelatinized starch enhances consistency of liquid medium at ambient temperatures. Therefore, it is suitable for instant foods production such as soups, baby foods, sauces, desserts, dry mixes, and cake powders. The use of this kind of starch in cake formulation improves dough consistency and strengths the role of gluten in dough (Majzoobi et al., 2011).

Biscuit is one of the popular cereal‐based confectionary products due to its availability, diversity, low prices, high shelf life, and energy content. Due to the existence of high protein content in standard products, phenylketonuria patients are not allowed to consume it. Removing the basic raw materials in biscuit formulations (wheat flour, egg whites, and dry milk) has profound undesirable effects on textural and qualitative characteristics of low‐protein biscuits.

Due to insufficient information about effect of modified starch on qualitative properties of low‐protein cereal‐based products, in present study, first, the qualitative characteristics of three types of corn starch (natural, pregelatinized, and cross‐linked) and the mixture of them were investigated. Then, their effects on quality of low‐protein biscuits were evaluated to understand the relationship of qualitative characteristics of starches and products made from them.

## 2 MATERIALS AND THE METHODS

### 2.1 Materials

Natural and modified starches (pregelatinized and cross‐linked) were provided from Glucosan Company (Tehran, Iran). Tara gum was obtained from Egzandal Corbe (America), and Lecithin and mono, diglyceride were purchased from Behpak companies (Behshahr, Iran) and Dalian (China), respectively. Sodium bicarbonate and ammonium were obtained from Kimia acid company (Tehran, Iran). Sugar and baking powder, vanilla, egg, and margarine butter were provided from local markets.

### 2.2 Qualitative properties of three types of starches

Thermal behavior of the starches was evaluated by Differential Scanning Calorimetry (DSC) (model DSC 200 F 3). The calibration of instrument was performed by silver and indium. Three mg of the samples was scanned in the thermal range of 30–320°C at the rate of 10°C/min (Wang, Rakotonirainy, & Padua, 2003).

The amylose content of samples was determined by the method of Williams, Kuzina, and Hlynka (1970). Briefly, 20 mg starch samples were weighted and 10 ml potassium hydroxide (0.5 N), 5 ml HCl (0.1 N), 0.5 ml of crystal iodine (0.2%), and potassium iodide (2%) solution were added to the samples and the volume made up with deionized water. The absorbance of solution was evaluated after 5 min at a wavelength of 625 nm using a UV‐Vis spectrophotometer (2100‐S model, UNICCO). Amylose content of the sample was quantified through a standard curve of pure amylose.

The appearance of starch granulates was evaluated by field emission scanning electron microscope (FE‐SEM—MIRA3TESCAN model). For this purpose, the samples were covered by a thin gold layer, and their appearance was investigated by an electronic microscope with an electrical potential of 5 kV (Haaj, Magnin, Pétrier, & Boufi, 2013).

### 2.3 Qualitative properties of combined starches

Water absorbability (Majzoobi et al., 2011) and gelatinization properties of the combined starches as follow in Table 1 were investigated by amylograph machine (Barabandar Company) (AACC‐22‐100).

**Table 1:** Starch composition of different treatments

Row	Natural starch	Pregelatinized starch	Cross‐linked starch
1	100	–	–
2	65	–	35
3	30	–	70
4	–	–	100
5	65	35	–
6	30	70	–
7	–	100	–
8	–	65	35
9	–	30	70

### 2.4 Biscuit preparation

To preparation of biscuit samples, egg yolk was mixed with sugar to form a creamy texture. Other ingredients such as vanilla, lecithin, and mono‐diglyceride were added to the cream and mixed thoroughly. Then, Tara gum was dissolved in distilled water and added to the mixture. Softened margarine was mixed thoroughly with residual sugar at ambient temperature. Afterward, mixed starch containing baking powder was added and mixed to it. Prepared solid and liquid phases were mixed to form a nonsticky soft batter with a light color. The amount of ingredients in batter formulation is represented in Table 2. The batter was stored for 0.5 hr at 4°C and shaped in circular form. Finally, the samples were cooked for 15 min at 220°C.

**Table 2:** Ingredients of the biscuit batter

Ingredients	Quantity (g)
Starch	200
Tara gum	1
Margarine	100
Lecithin	0.5
Mono‐diglyceride	1
Sugar	100
Baking powder	4
Vanilla	0.5
Water	40
Yolk	20

### 2.5 Qualitative properties of the biscuit samples

Textural properties of biscuit samples were analyzed using a Texture Analyzer (CT3 model, Brookfield Company) after storage at 20°C for 24 hr. The samples were fixed in 3 cm distance, and the brittleness in central part of them was investigated with a knife‐edge probe (60 mm width and 3 mm thickness) in speed of 0.8 mm/s (Savitha, Indrani, & Prakash, 2008).

Density of the biscuits was determined by rape seed displacement method, and their diameters were evaluated by digital caliper (Savitha et al., 2008). The moisture content of samples was measured based on the standard method of AOAC (International, 2005). The transparent solution of 10 g biscuit in 100 ml of distilled water (20 min after mixing) was used for pH measurement with digital pH meter (Iran national standard, number 37). Sensory properties of the biscuits like color, taste, smell, crispness, and total acceptance were evaluated by 13 children suffering Phenylketonuria and also their parents. According to the 5‐point Hedonic method, 1 and 5 belonged to the least and the most desirable samples in each target, respectively. The best sample was selected, and its qualitative properties were analyzed carefully.

#### 2.5.1 Physicochemical properties of the best sample

Total ash and protein content of the selected samples were measured based on standard the methods of AOAC (International, 2005). Peroxide value (milliequivalents oxygen/kilogram of extracted fat) was also determined as described by the official methods of European Communities 2568/91.

Reverse‐phase high‐performance liquid chromatography (HPLC) was employed for determining the amino acids profile (Perkin‐Elmer). 0.2 g of defatted sample using chlorophyll and methanol was hydrolyzed by HCL (6 M) for 24 hr at 110°C. HCL was removed in rotary at 45°C, and dried sample was prepared for the amino acid derivation by phenyl isothiocyanate. 1–10 µl of the samples was injected to C18 column (9.3 mm × 300 mm inner diameter). A standard solution containing amino acids (Arg, Ala, Thr, His, Gly, Ser, Gly, Asp, Tyr, Pro, Phe, Leu, Ilu, Cys, Met, Val, and Lys) was prepared. The amino acids were identified by retention time comparison with authentic standards. Quantification was performed on the basis of the standard method (basing the regression on the ratio of the analytic response to that of the standard) using fluorescence signals at a wavelength of 254 nm. Chromatographic data were analyzed using a Breeze Controller software (version 3.200, Waters, and Milford, MA, USA) (Matloubi, Aflaki, & Hadjiezadegan, 2004).

### 2.6 Statistical analysis

Statistical analysis of the data was performed (ANOVA) according to completely randomized design followed by LSD's test to compare the means of the dependent variables at significant probability level through the statistical software version 9 (SAS). The correlation coefficients of parameters were investigated based on Pearson test.

All the measurements were carried out in triplicates, and the results were presented as mean ± standard deviation (*SD*). Diagrams were constructed using the Microsoft Excel 2010 software.

The data derived from a sensorial experiment with the certainty of 95% were analyzed and compared by variance analysis of Kruskal–Wallis test.

## 3 RESULTS AND DISCUSSIONS

### 3.1 Qualitative properties of starches

**Figure 1:**
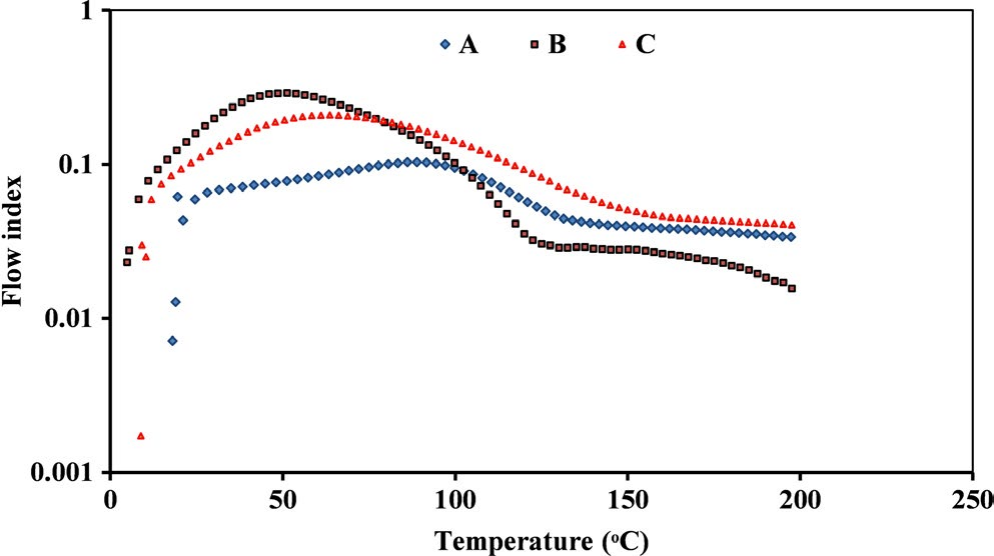
Thermogram of three types of starch

The qualitative properties of three kinds of starches are presented in Table 3. Thermal curve of samples had only one exothermic peak (Figure 1). Comparatively, the value of *T_g_* in cross‐linked starch was considerably higher than natural and pregelatinized starches. Cross‐linking process usually decreases starch chains mobility and increases required energy for modifying physical properties and transition temperature of samples in heating process (Chung, Woo, & Lim, 2004). Quality and chemical properties of starch molecules are affected by modification process due to destruction or connection of starch chains. Therefore, interaction of linear chains and partial crystallinity of starch affect *T_g_* of granules (Liu et al., 2010).

**Table 3:** Qualitative features of three types of starch

Starch	Glass transition temperature (°C)	Low‐branched chains (amylase) (%w/w)
Natural	39.35^b^	27.43^c^
Cross‐linked	78.65^a^	34.23^b^
Pregelatinized	39.90^b^	37.55^a^
LSD	1.88	1.00

Note

Values are presented as mean ± *SD* (*n* = 3).

Values followed by the same letter, within the same column, were not significantly different (*p* < .05).

There were considerable differences in the content of low‐branched chain in three types of starch (Table 3). Maximum and minimum content of low‐branched chain was observed in pregelatinized and natural starch, respectively. It is due to effect of heating process, pH changes, and other treatments in modification processes.

**Figure 2:**
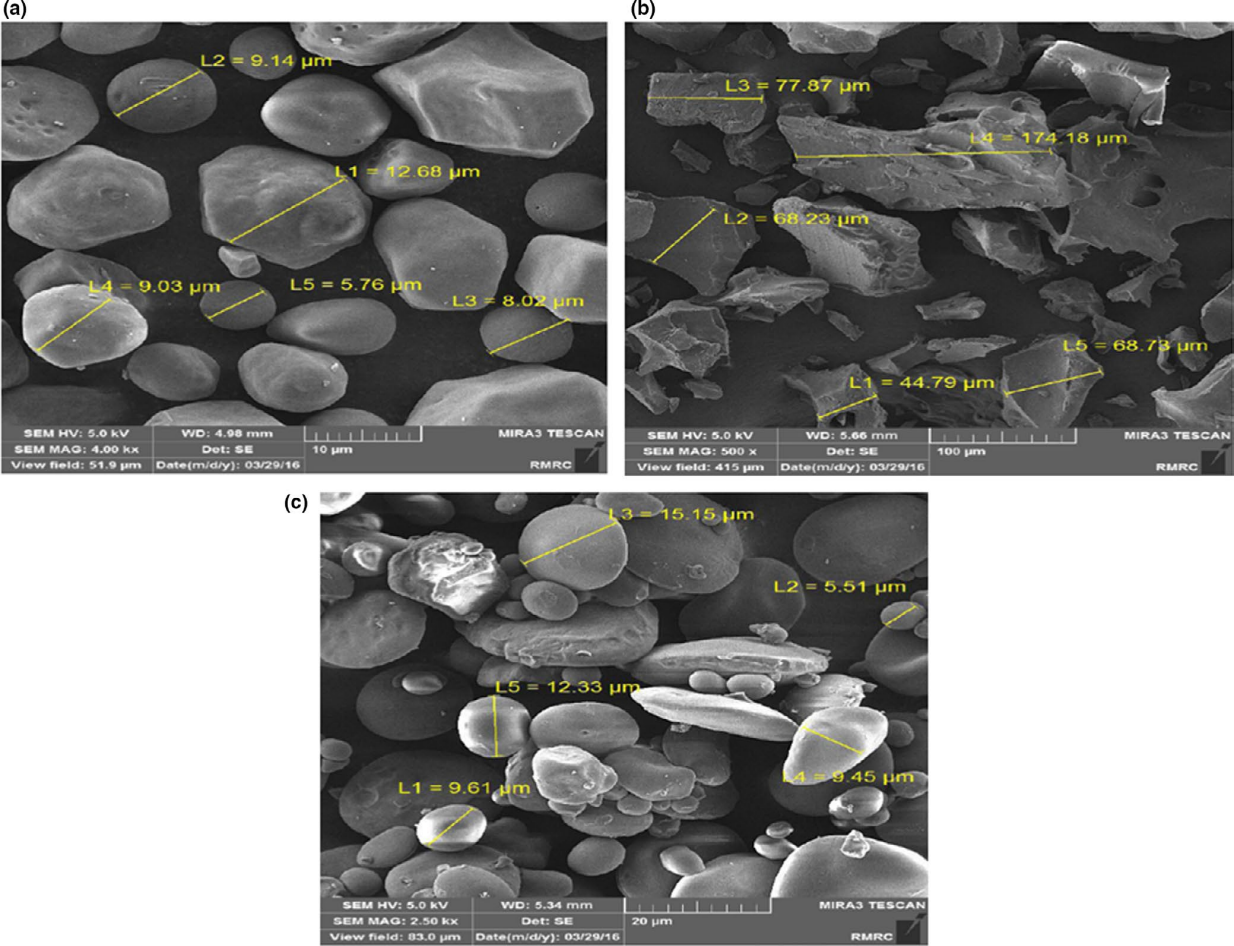
SEM images of (a) natural (b) pregelatinized (c) cross‐linked starches

The SEM images of natural and modified starch granule are depicted in Figure 2. In natural starch, typical homogenous granules in dimension of 10–15 μm were observed. In pregelatinized starch, the original appearance of granules was changed due to the primary heating and cooling of granules in water. These particles were 3–30 μm. Different large and small granules in cross‐linked starch were observed, and the uniformity of granules was lower than other types. The external surface of these granules was round, and their dimension was in the range of 5–15 μm. Aggregation of starch chains in heating process and chemical modification leads to rearrangement of granule structure and creates widespread dimension of them (Carmona‐Garcia, Sanchez‐Rivera, Méndez‐Montealvo, Garza‐Montoya, & Bello‐Pérez, 2009).

### 3.2 Qualitative properties of mixed up starches

**Figure 3:**
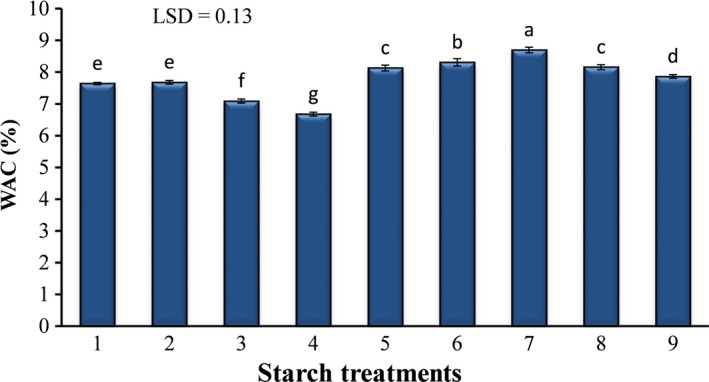
Water absorption capacity of mixed starch samples. Starch composition of different treatments are shown in Table 1. Values are presented as mean ± *SD* (*n* = 3). Values followed by the different letters were significantly different (*p* ≤ .05), according to LSD's test

The water absorbability of starches in different treatments is shown in Figure 3. The maximum and minimum water absorptions were observed in the samples containing 100% pregelatinized and 100% cross‐linked starches, respectively. With increasing cross‐linked starch, a decrease in the water absorption capacity of samples was observed, while pregelatinized starch increased this feature in a mixed starch significantly (*p* ≤ .05). Cross‐linking process provides stronger interaction among starch chains and consequently increases their resistance against water absorption (Miyazaki, Hung, Maeda, & Morita, 2006). Destruction of starch granules and reduction of their crystallinity in the gelatinization process, which was confirmed in SEM images, improve water absorbability of pregelatinized starch.

Gelatinization characteristics of mixed starches are presented in Table 4. The results showed that by increasing the amount of pregelatinized starch in the samples, the maximum viscosity of paste, starting and finishing temperature of gelatinization, and the differences between them decreased when compared to control sample. It is related to the gel form ability of pregelatinized starch at environment temperature due to the destruction of granules structure in the primary gelatinization process. Reducing the length of starch chains during heating process usually decreases water absorbability of starch and viscosity of the produced gel. Therefore, in gelatinized starch, the starch chains need lower energy for being inflated because of weak connections among molecules (Nakorn, Tongdang, & Sirivongpaisal, 2009).

**Table 4:** Gelatinization properties of mixed starches

Starch	Start of gelatinization (°C)	Maximum viscosity (cP)	End of gelatinization (°C)
1 (Control)	63.93^a^	4,094.33^a^	74.10^f^
2	67.43^ab^	3,414.00^d^	77.63^e^
3	63.63^c^	3,427.67^c^	88.27^b^
4	61.10^d^	2,622.67^f^	92.17^a^
5	65.67b^c^	3,904.33^b^	83.83^d^
6	66.10^ab^	1,863.33^g^	70.60^g^
7	30.43^g^	923.67^i^	31.83^i^
8	51.77^f^	1,696.67^h^	67.37^h^
9	58.17^e^	2,962.33^e^	85.70^c^

Note

Starch composition of different treatments is shown in Table 1.

The end of gelatinization temperature and the difference between the start and the end point of gelatinization were notably higher in the samples containing cross‐linked starch than control.

This observation is associated with more powerful structure of granules in this starch due to the difference in type and strength of linkages among molecules which increase required energy for the destruction of starch chains (Miyazaki et al., 2006).

According to Table 4, the maximum and minimum of gelatinization temperatures were related to the samples containing 100% cross‐linked and 100% pregelatinized starch, respectively. Additionally, it was found that the maximum viscosity in the sample comprising pregelatinized and cross‐linked starches was lower than that of control. The lowest viscosity was related to the sample containing 100% pregelatinized starch. Low viscosity in the samples containing cross‐linked starch is due to the interactions and regularity of starch chains. Long heating process because of great difference between the start and final of gelatinization temperature of cross‐linked starch with destruction of chains and aligning them to direction of stirerr axis decreases gel viscosity (Jyothi, Moorthy, & Rajasekharan, 2006).

### 3.3 Physicochemical and sensorial properties of biscuits

#### 3.3.1 Moisture content

**Figure 4:**
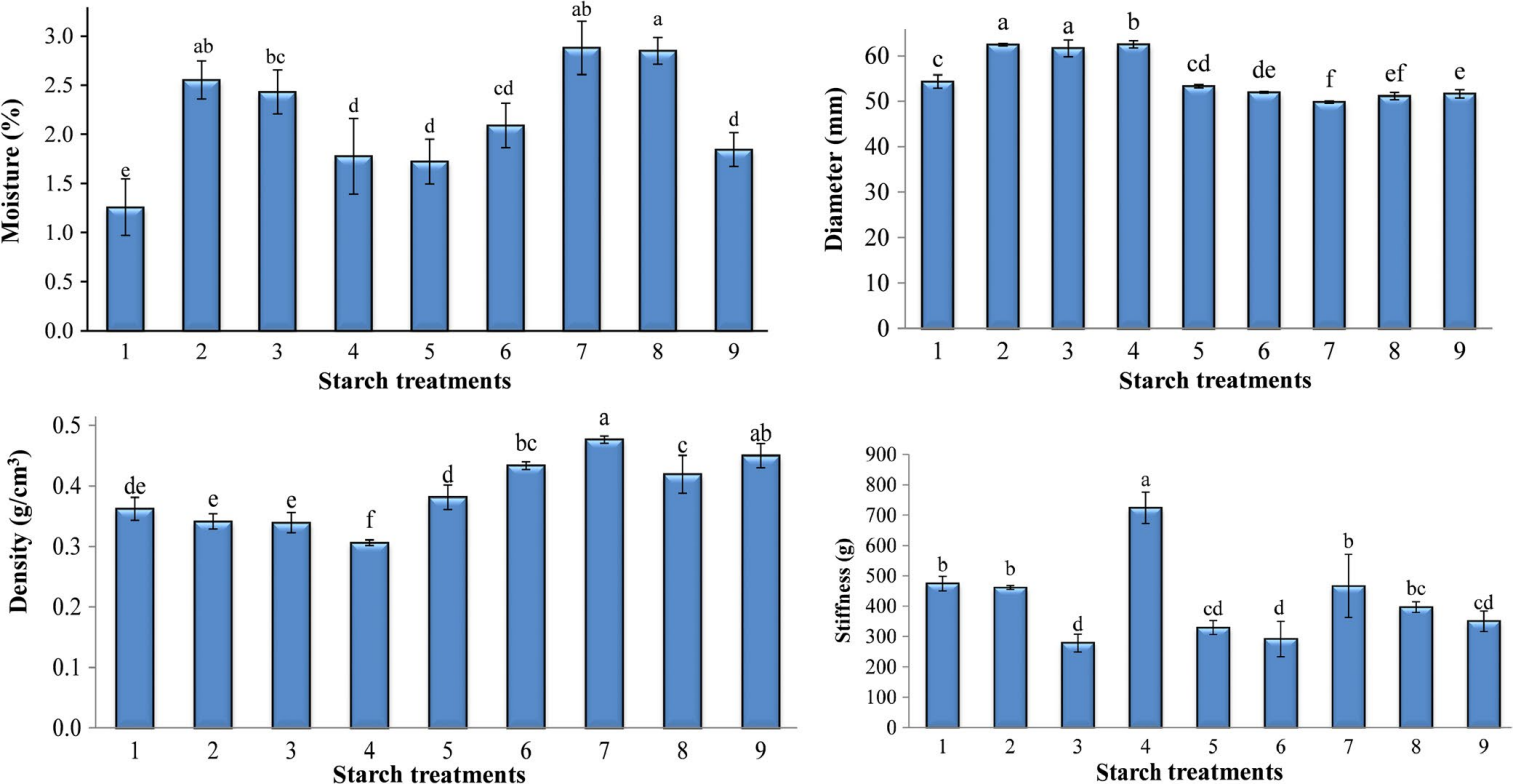
Effect of starch composition on moisture, dimension, density, and stiffness of biscuit. Averages (± standard deviation) with the same letters in 5% level of LSD test do not have significant differences. Starch composition of different samples has been shown in Table 1

As presented in Figure 4, modified starch specially pregelatinized starch increased the moisture content of biscuits than control due to high water absorption capacity. The maximum and minimum moisture contents were related to the samples comprising 100% pregelatinized and 100% natural starch (control), respectively. Cross‐linked starch is more robust against water absorption, but it has more ability in protection of absorbed water in heating process (Miyazaki et al., 2006). Therefore, in the biscuits containing cross‐linked starch, lose of water in the heating process was more difficult and moisture content of them preserved. It should be noted that the moisture content of the samples in this project was matched to the standard of this product (44‐15 AACC, 1999).

#### 3.3.2 Biscuits diameter

Starch compositions had also significant effect (*p* ≤ .05) on product diameter (Figure 4b). Minimum and maximum diameters of biscuit were observed in the samples containing 100% pregelatinized and 100% cross‐linked starches, respectively. High viscous and strong dough due to the presence of flour and other components with too tight connections to water produces products with small size and volume (Ordorica‐Falomir & Paredes‐Lopez, 1991). High viscosity in dough prevents the growth and expansion of water vapor and carbon dioxide bubbles from the leavening agent and restricts the volume of biscuits (Mancebo, Picón, & Gómez, 2015). In the presence of cross‐linked starch with minimum water absorbability, free water decreased dough viscosity, and therefore, an average diameter of the product increased. Furthermore, high water absorbability of pregelatinized starch at ambient temperature produced a gel‐like structure that deformed the shaped dough and reduced the product diameter significantly (*p* ≤ .05) (Tavares et al., 2016).

#### 3.3.3 The density of biscuits

The starch composition had a significant effect on density of products (Figure 4c). According to the results, by increasing the quantity of pregelatinized starch, the density of product increased; while in the present of cross‐linked starch, the density of biscuit decreased significantly (*p* ≤ .05). Therefore, the samples containing 100% cross‐linked and 100% pregelatinized starch had minimum and maximum density, respectively. High water absorbability of starch reduced expansion rate of product in heating process. Therefore, maximum density was observed in the biscuit contain maximum amount of starch with the highest water absorbability. Increasing the viscosity of dough containing pregelatinized starch at ambient temperature also effectively reduced air bubbles release and increased product density during heating process (Mancebo et al., 2015). It is also related to gelatinization properties of starch granules and the amount of gelatinized starch in the product (Demirkesen, 2016).

#### 3.3.4 Textural properties

The stiffness of product decreased at low substitution levels of modified starch. In complete replacement of natural starch with modified starch, especially cross‐linked one, biscuits stiffness increased significantly (*p* ≤ .05) (Figure 4d). Therefore, maximum stiffness observed in the sample containing 100% cross‐linked starch. The stiffness of biscuit is mainly depend on different factors such as quantity and quality of protein, the starch properties, the total ash, fiber and moisture contents of the product, and also the production process. Increasing moisture content of biscuit will make the texture of product softer. Therefore, constituents of product and production process should be considered as effective parameters on texture of product. Difference in gelatinization temperature and degree of gelatinized starch before textural setting of product, especially in the presence of limited moisture content also influence qualitative properties of biscuit texture. Density and stiffness of product usually increase at lower degree of starch gelatinization. Reducing starch granules gelatinization of cross‐linked starch due to high interaction among amylose and amylopectin is effective parameter on increasing biscuit hardness (Demirkesen, 2016).

Pearson correlation coefficients of starch specifications and quality attributes of the product are summarized in Table 5. Average dimension and stiffness of biscuit had significant negative correlations with density of products. Effect of density on dimension of product changes was higher than that observed for stiffness. Effect of water and dough compounds interactions in increasing dough viscosity and reducing expansion of steam bubbles and CO_2_ are an effective parameters in reducing volume of biscuit (Mancebo et al., 2015). Increasing size and dimension of biscuit during heating process makes the structure of product more porous and decreases biscuit density. On the other hand, there were significant negative correlations among water absorption capacity and the amylography parameters (starting and ending temperature of gelatinization and the maximum viscosity of starch paste). It also was found that amylography parameters had significant positive correlations to each other. By increasing the water absorption capacity of starch, start and end of gelatinization occurred at low heating conditions as well as the viscosity of produced gel reduced. Therefore, the temperature difference between start and end of the gelatinization process is associated with starch resistance against water absorption (Singh, Kaur, & McCarthy, 2007). Increasing time differences between the beginning and the end of gelatinization temperature usually enhance starch paste viscosity. Temperature difference between the beginning and the end of gelatinization is related to distribution and nonuniformity of granules sizes. Typically, larger granules have lower start and final temperature of gelatinization than smaller starch granules due to higher central particle density of smaller granules and better penetration of water to larger ones (Orsuwan & Sothornvit, 2015). There were significant negative correlations between water absorbability of starch, stiffness, and average diameter of product, whereas a significant positive correlation observed between water absorption capacity and density of samples. The interaction of water and compounds in biscuits increases dough viscosity and product density as a results of reducing expanding rate, size, and volume of bubbles (Mancebo et al., 2015). Amylograph properties of starch had significant positive correlations with average dimension of product, and significant negative correlation with moisture content and density of biscuits. At lower amylography specifications (start and final gelatinization temperature and maximum viscosity), easier degradation and water absorption of starch granules increased viscosity of batter at room temperature. However, gelatinization temperature of starch granules usually increases at high sugar content in biscuit batter. Distribution and growing up steam bubbles and CO_2_ obtained from chemical leavening agents decrease in high viscosity batter. It reduces product dimensions and induces biscuit density increment consequently (Demirkesen, 2016). In the starch samples studied in the present project, pregelatinized starch had the lowest amylography specifications and the highest water absorbability than others.

**Table 5:** Pearson correlation coefficients of starch specifications and quality attributes of biscuit

	Stiffness	Moisture	Density	Diameter	Water absorption capacity	Start of gelatinization temperature	Maximum viscosity	End of gelatinization temperature
Stiffness	1							
Moisture	−0.180	1						
Density	−0.389*	0.311	1					
Diameter	0.098	0.032	−0.774**	1				
Water absorption capacity	−0.441*	0.331	0.841**	−0.678**	1			
Start of gelatinization temperature	−0.134	−0.560**	−0.609**	0.524**	−0.514**	1		
Maximum viscosity	−0.081	−0.628**	−0.617**	0.545**	−0.500**	0.787**	1	
End of gelatinization temperature	0.026	−0.498**	−0.699**	0.523**	−0.764**	0.822**	0.708**	1

Note

*, ** are meaningful in statistical levels 0.05 and 0.01, respectively (*n* = 27).

#### 3.3.5 Sensory properties

Table 6 shows the effect of starch compositions on sensory attributes of the samples like appearance, taste, texture, after taste, and general reception. As shown in Table 6, the minimum and maximum scores of apparent attributes were related to control and sample containing 30% pregelatinized and 70% cross‐linked starches, respectively. Color is one of the most important effective factors on consumer acceptance. Difference in natural color of product components and their reaction during heating process affect color properties of product. Color changing of biscuit is mainly occurred due to Maillard and Caramelization reactions. Partial hydrolysis of glycoside bonds in starch molecules during modification process produces regenerative sugars that react in browning reactions. Cracks on the biscuit surface and nonuniformity of products thickness are the main reasons in reducing final products score specially in control sample. The lowest scores of taste and tenderness were observed in control and sample containing 30% typical starch and 70% cross‐linked starch, respectively. Furthermore, the sample containing 100% cross‐linked starch and control had the lowest scores in term of taste and general acceptability. Consequently, in low‐protein content biscuits, the sample with 30% pregelatinized starch and 70% cross‐linked starches had the highest significant scores in five sensory attributes including apparent, taste, tenderness, after taste, and general acceptability.

**Table 6:** Effect of starch composition on sensorial characteristics of biscuits

Treatments	Appearance	Taste	Texture	After taste	General acceptability
1 (control)	1.5^e^	1.90^e^	2.55^c^	2.25^e^	2.20^e^
2	1.6^e^	1.9^e^	2.65^c^	1.90^ef^	2.40^e^
3	2.1^d^	1.90^e^	2.55^c^	1.55^fg^	2.85^d^
4	3.2^5^	2.45^d^	3.70^b^	1.45^g^	3.10^cd^
5	31^c^	2.70^cd^	3.35^b^	3.35^cd^	3.50^bc^
6	3.3^bc^	3.05^c^	2.75^c^	3.60^bc^	3.25^bcd^
7	3.30^bc^	3.45^b^	2.25^c^	3.05^d^	3.15^cd^
8	3.50^b^	3.60^b^	2.50^c^	3.8^b^	3.70^b^
9	4.80^a^	4.25^a^	4.4^a^	4.55^a^	4.8^a^

Note

In each column, averages with similar letters do not have meaningful differences in 5% level.

Starch composition of different treatment has been shown in Table 1.

#### 3.3.6 Protein content

The protein content of the selected sample with 30% pregelatinized and 70% cross‐linked starch was 1.27 ± 0.15 g per 100 g biscuit. Minimum acceptable range of biscuit protein according to Iran National Standard (number 37) is 10 g per 100 g of the sample. Therefore, about 85%–90% reduction in protein content of the selected sample was achieved. By considering the acceptable limit of daily proteins (50–65 g) for PKU, one serving of this produce (about 30–50 g) supplies only 1% of the daily consumable protein of patient. Peroxide index, ash content, and pH of this sample were 0.06 ± 0.02 (mg oxygen/kg of fat), 1.05 ± 0.05 (g/100 g of product) and 6.8 ± 0.3 which were in an acceptable range according to standard qualify attributes of this product (Iran national standard, number 37).

Amino acids profile of the selected sample is presented in Table 7. Seventeen amino acids were identified in this product. There was about 50 mg phenylalanine in 100 g of biscuit. Therefore, in a consummation term, in each serving of this product, 15–35 mg phenylalanine that supplies about 5%–10% of the allowable daily consummation of this amino acid for Phenylketonuria patients will be received (300–750 mg/day) (Yaseen, 2011).

**Table 7:** Amino acids profile of the low‐protein biscuit.

Amino acids	Milligram/100 g of sample
Glu	13
Asp	180
Ser	92
Gly	39
His	<26
Arg	66
Thr	55
Ala	60
Pro	115
Tyr	90
Val	173
Met	157
Cys	<26
Ilu	37
Leu	90
Phe	50
Lys	3

## 4 CONCLUSION

Modification of starch had specific effects on its properties such as the granules appearance in microscopic imaging, thermal properties such as glass transition temperature and gelatinization characteristics, and water absorption capacity. Specification of starch had considerable effects on the qualitative properties of low‐protein biscuit. Cross‐linked starch reduced viscosity and induced heat stability of granules during product baking in comparison to natural starch. The best composition of starch‐based on the qualitative attributes of the products with the highest sensorial acceptability was the mixture of 70% cross‐linked and 30% pregelatinized starches. This product with 1.27 g protein (12 mg phenylalanine) in 100 g biscuit is introduced as low‐protein biscuit and a safe product for Phenylketonuria disease. It was found that in each serving of low‐protein biscuit formulated by modified starches, 15–35 mg phenylalanine that supplies about 5%–10% of the allowable daily consummation of this amino acid for Phenylketonuria patients will be received (300–750 mg/day). Thus, a diet containing the developed biscuit can help to the care of Phenylketonuria patients.

## CONFLICT OF INTEREST

The authors declare no conflict of interest.

## ETHICAL APPROVAL

This article does not contain any studies with human participants or animals performed by any of the authors.
